# Improved Exponential and Cost-Weighted Hybrid Algorithm for Mobile Robot Path Planning

**DOI:** 10.3390/s25082579

**Published:** 2025-04-19

**Authors:** Ming Hu, Shuhai Jiang, Kangqian Zhou, Xunan Cao, Cun Li

**Affiliations:** School of Mechanical and Electronic Engineering, Nanjing Forestry University, 159 Longpan Road, Nanjing 210037, China; 18115858931@163.com (M.H.); 15261862617@163.com (K.Z.); 18934515584@163.com (X.C.); lc010130@163.com (C.L.)

**Keywords:** hybrid algorithm, dynamic window approach, path planning, navigation

## Abstract

The A* algorithm is widely used in mobile robot path planning; however, it faces challenges such as unsmooth planned paths, redundant nodes, and extensive search areas. This paper proposes a hybrid algorithm combining an improved A* algorithm with the Dynamic Window Approach. By quantifying grid obstacle data to extract environmental information and employing a grid-based environmental modeling method, the proposed approach enhances path smoothness at turns using second-order Bezier curve smoothing. It improves the heuristic function and child node selection process, applying these advancements in experimental path planning scenarios. A simulated 2D map was constructed using point cloud scanning in RViz to validate the hybrid algorithm through simulations and real-world outdoor tests. Experimental results demonstrate that, compared to the A* and DWA algorithms, the improved hybrid algorithm enhances search efficiency by 10.93%, reduces search node count by 32.26%, decreases the number of turning points by 36.36% and the value of turning angle by 34.83%, shortens the total path length by 22.05%, and improves overall path smoothness. Simulations and field tests confirm that the proposed hybrid algorithm is more stable, significantly reduces collision probability, and demonstrates its applicability for mobile robot localization and navigation in real-world environments.

## 1. Introduction

In the field of mobile robot applications, path planning has become a core task. Traditional delivery robots operating in known environments rely on sensing systems to collect environmental data [1], calculate navigation parameters, and track pre-defined paths to navigate safely while adhering to kinematic and dynamic constraints. In environments with numerous obstacles, mobile robots often face challenges such as slow convergence and low accuracy, making effective path planning difficult [2]. Designing optimized algorithms tailored to these scenarios can significantly enhance the robot’s performance and efficiency. By incorporating the heuristic function of the A* algorithm, mobile robots can efficiently and quickly determine the optimal path to reach target points. However, in complex environments, addressing the issue of local optima is particularly critical. Mobile robots must perform global path planning to identify a safe and optimal route from the starting point to the destination and use local path planning to avoid obstacles. Key planning metrics include navigation distance accuracy and angle precision, making the integration of global and local path planning an effective solution for autonomous navigation. The combined A* and DWA algorithm, known for its stability and innovation potential, continues to lead to advancements in integrated path planning.

In fact, numerous researchers have developed successful path planning methods to avoid obstacles. In the context of planning collision-free routes and enabling autonomous navigation for unmanned aerial vehicles, Yang [3] proposed an improved rapidly exploring random tree algorithm to address the challenges of obstacle space planning. This approach incorporates ant colony optimization to select superior extension points based on the pheromone concentration associated with spatial obstacles. Through multiple iterations, the path converges to an optimal solution that satisfies constraints on continuity and output stability. To tackle the challenge of avoiding dynamic underwater obstacles and to address the suboptimality in path planning for underwater vehicles, Li et al. [4] integrated an enhanced A* algorithm with model predictive control. They developed a six-degree-of-freedom motion dynamics model for underwater vehicles, enabling the system to dynamically adjust the planned route in real time when encountering unknown moving obstacles, thus ensuring effective obstacle avoidance. In the domain of real-time path planning applications with high computational demands, Ntakolia et al. [5] designed an intelligent assisted navigation system. This system leverages a lightweight deep convolutional neural network, a fuzzy planning algorithm, and a novel fuzzy logic-based ant colony optimization algorithm for obstacle detection and avoidance. This approach significantly enhances the computational efficiency and capability of path planning, making it well-suited for real-time operational environments.

The A* algorithm, recognized as a classic path planning method, excels in computing globally optimal paths with a straightforward programming model for obstacle avoidance. In many real-world engineering applications, the traditional A* algorithm often fails to meet contemporary performance expectations, exhibiting problems such as excessive node traversal, too many turning points, and non-uniform path distribution. To address these issues and better match practical requirements, researchers have proposed a variety of cutting-edge improvements. Wang et al. [6] introduced the EBS-A* algorithm, which integrates extended distance, bidirectional search, and smoothing into the traditional framework, thereby reducing the number of path nodes and right-angle turns—albeit at the cost of greater computational overhead due to the bidirectional search. Meanwhile, Liao et al. [7] tackled challenges such as high node traversal counts, lengthy search times, and limited suitability for dynamic environments by proposing an adaptive A* algorithm. This approach incorporates adaptive weighting into the A* heuristic function and uses a refinement algorithm to remove redundant points, effectively reducing node traversal and runtime, shortening path length, and improving efficiency. However, its performance in narrow spaces remains less than ideal. Zhang et al. [8] introduced a bidirectional search strategy and an enhanced evaluation function to reduce the total number of traversed nodes, smooth the path by eliminating turning points, and improve search efficiency. In complex environments, Li et al. [9] proposed a backend-optimized hybrid A* algorithm with an adaptive node expansion strategy to handle environmental complexity. They employed quadratic programming to smooth discrete path points, ensuring Seamless and smooth continuous transitions in heading and speed, thereby enabling rapid responses to environmental changes and improving planning efficiency. However, Zhang and Li’s enhancement of the A* algorithm restricts path smoothing to the simulation phase, without considering the motion parameter characteristics of mobile robots. Consequently, the smoothing efficiency during robot turns lacks practical reliability, limiting its effectiveness and applicability in large-scale, complex real-world environments.

Single-path planning metrics are insufficient to meet robot operational needs, necessitating the inclusion of local path planning methods to resolve local optima. Xu et al. proposed an improved A* algorithm and local map scaling technique to enhance global path precision and optimization [10]. By incorporating an estimated unknown path cost function and using an improved LQR method for trajectory tracking, they achieved a 23% reduction in time and a 21% decrease in path length. Zeng et al. improved the A* algorithm’s heuristic function by introducing dynamic weighting with Floyd’s algorithm to remove redundant points [11]. Dynamic weighting coefficients from the DWA algorithm were incorporated to adjust weights based on obstacle distribution, ensuring the robot’s safe arrival at its target. Chen et al. [12] integrated the A* and DWA algorithms to solve unmanned path planning challenges by incorporating the dynamic window method into the evaluation subfunction in complex environments, enhancing path planning safety. The integrated algorithm supports the addition of multi-strategy approaches, enabling mobile robots to more effectively identify dynamic and static obstacles, thereby improving path planning efficiency. Kumar et al. [13] introduced a novel fusion of WOA (Whale Optimization Algorithm) and fuzzy logic, achieving a 20.63% optimization in path length, while Akay et al. [14] enhanced the sine–cosine algorithm to address multi-robot path planning in environments with dynamic and static obstacles. Sheng et al. [15] proposed a multi-level hybrid A* algorithm to address narrow unstructured channel issues, creating feasible sub-paths for each boundary point and optimizing speed profiles for improved layered trajectory planning speed. Meng et al. [16] presented a hybrid A* algorithm with safety-enhanced designs, incorporating multi-phase dynamic optimization strategies to divide path planning into multiple stages for processing and dynamic optimization, significantly improving search efficiency in time and space. Dang et al. [17] refined motion primitives during the forward search phase, eliminating the need for complex path smoothing techniques, resulting in smoother curves. During the analytic expansion phase, they introduced an improved Reeds–Shepp method, allowing for multiple turning radii and curvatures, which reduced collision risk costs by approximately 20%.

The structure of this article is as follows: The second section mainly improves the traditional A* algorithm, making unified improvements from three aspects: the heuristic function, the neighborhood search strategy, and the curve optimization. These improvements can shorten path lengths, boost search efficiency, and reduce the number of turning points compared to the conventional approach. Nonetheless, merely refining the A* algorithm is not enough to allow mobile robots to adapt effectively to dynamic environments or guarantee stable path planning. In Section 2.3, the DWA algorithm is introduced as the local path planning algorithm, improving the path without adding computational complexity and ensuring efficient path search performance. Section 3 presents experiments of the fusion algorithm in both simulated and real environments. The experiments showcase the construction of the hardware model of a mobile robot equipped with a 2D lidar, and the Cartographer algorithm is employed for environmental mapping. In the simulation environment, comparative tests of the fusion algorithm demonstrate the superiority of the improved algorithm. Finally, tests in the actual environment demonstrate that the application of the fusion algorithm meets the expected outcomes.

Additionally, by integrating the DWA algorithm for local path planning, the approach achieves shorter, smoother paths without increasing computational complexity, thereby ensuring efficient path search and effective dynamic obstacle avoidance. Finally, the hybrid algorithm is tested in a robot environment, with the results demonstrating that the accuracy of the mobile robot’s autonomous navigation system meets the expected performance criteria.

## 2. Improvement of A* Algorithm

### 2.1. Basic Law of Traditional A* Algorithm

The A* algorithm is a heuristic search method designed to find the optimal path. It combines the depth-first characteristics of Dijkstra’s algorithm [18] with the breadth-first characteristics of the greedy algorithm [19]. By using an evaluation function to determine the robot’s search direction, A* generates a path with the minimal total cost [20]. Its heuristic function is expressed as follows:

(1) f(n)=g(n)+h(n)

f(n) represents the evaluation function for reaching the goal from any node n; g(n) represents the actual cost from the start node to n, and its value is calculated as shown in Equation (2); and h(n) represents the estimated cost from n to the goal node. Common methods for computing h(n) include the Euclidean distance and Manhattan distance, as shown in Equation (3).

(2) $$g(n)=\sqrt{{\left(x_{n}-x_{0}\right)}^{2}+{\left(y_{n}-y_{0}\right)}^{2}}$$

(3) $$h(n)=\left\{\begin{array}{c} \sqrt{{\left(x_{g}-x_{n}\right)}^{2}+{\left(y_{g}-y_{n}\right)}^{2}}\left(Euclidean\right)\\ \left|x_{g}-x_{n}\right|+\left|y_{g}-y_{n}\right|\left(Manhattan\right) \end{array}\right.$$

Let $\left(x_{n},y_{n}\right)$ denote the center coordinates of a moving node in the global map, and denote the center coordinates of the goal node $\left(x_{g},y_{g}\right)$. The coordinates of the current start node $t_{0}$, the current node t, and the goal node $t_{1}$ are represented as $\left(x_{0},y_{0}\right)$, $\left(x_{t},y_{t}\right)$, and $\left(x_{1},y_{1}\right)$, respectively [21].

### 2.2. Ways to Improve the A* Algorithm

#### 2.2.1. Improve the Heuristic Function

In the A* algorithm, the Manhattan distance is used as the default heuristic function, as shown below:

(4) $$h(n)=\left|x_{g}-x_{n}\right|+\left|y_{g}-y_{n}\right|$$

Manhattan distance calculates the sum of the absolute differences between the coordinates of two points, making the computation straightforward. However, it is primarily suited for horizontal or vertical movements between grid points, limiting the robot’s flexible movement within a grid cell in multi-dimensional spaces. Accordingly, to balance the trade-off between search speed and path length, employing a weighted Euclidean distance as the heuristic function is an effective approach [22], which can yield a globally optimal route while maintaining overall acceptability. However, in complex environments, the heuristic may not fully exploit cost information and terrain features, leading to less efficient paths. A creative solution is to integrate an advanced cost-weighted formula into the heuristic; by carefully considering cost data and map characteristics, and by fine-tuning the weighting values, deviation from the optimal solution can be minimized. Let the Euclidean function be denoted as d, as shown in Equation (5). In this formulation, the Euclidean distance is weighted first, and then the average cost is incorporated, as shown in Equation (6). By combining cost matrix information with an adaptive adjustment of the heuristic function, a more balanced trade-off between path length and cost can be achieved, thereby facilitating the discovery of the shortest path. This approach is especially effective in environments with dense obstacles or pronounced cost disparities.

(5) $$d=\sqrt{{\left(x_{g}-x_{n}\right)}^{2}+{\left(y_{g}-y_{n}\right)}^{2}}$$

(6) $$h(n)=\omega (n)\cdot d+k\cdot d_{0}\cdot cost_{avg}$$

k is the cost weighting coefficient, determined based on the map dimensions and cost settings, in scenarios where the map exhibits significant cost variations, increasing the k value is advisable; conversely, if distance is the primary concern, the k value should be decreased. $cost_{avg}$ represents the average cost of the route from the current point to the target, and $d_{0}$ signifies the distance factor for the Euclidean distance.

Conversely, as shown in Equation (6), $\omega \left(n\right)$ denotes the weighting factor. When the weight ω(n) is small, the search becomes slower, allowing more time to find the optimal path. When $\omega \left(n\right)>1$, the heuristic accelerates the search at the expense of optimality, whereas a value of ω approaching one yields near-optimal results, with improved search speed, but may result in suboptimal paths. To mitigate this issue, a segmented weighting strategy can be adopted: using exponential weighting in the middle segments of the path and constant weighting in the start and end segments. This approach effectively balances search time and path optimality. The specific formulas are shown in Equations (7) and (8):

(7) $$\omega \left(n\right)=C,n\in \left(0,n_{1}\right)\cup \left(n_{2},n_{end}\right)$$

(8) $$\omega \left(n\right)=\left(1.5+h\left(n\right)/g\left(n\right)\right)\cdot e^{h\left(n\right)},n\in \left(n_{1},n_{2}\right)$$

In the function of the aforementioned two equations, the entire search process can be divided into three segments: the initial interval $\left(0,n_{1}\right)$, the middle interval $\left(n_{1},n_{2}\right)$, and the final interval $\left(n_{2},n_{end}\right)$. In this context, n denotes the node under examination at any given moment. As the search proceeds, $\omega \left(n\right)$ has been changed as n grows. Meanwhile, $g\left(n\right)$ signifies the actual cost from the start node to the current node, whereas $h\left(n\right)$ indicates the predicted cost from the current node to the target node $n_{end}$. By taking both the actual cost and the predicted cost into account, one can determine the most efficient shortest path.

ω(n) represents the weighting factor, which is dynamically adjusted based on the size of the map, the complexity of the environment, and the processing capability of the algorithm’s processor. By introducing this weight coefficient, the search efficiency and path accuracy can be balanced to a certain extent. In different application scenarios, dynamically adjusting this weight coefficient is crucial for enhancing the overall performance of the algorithm.

**Figure 1:**
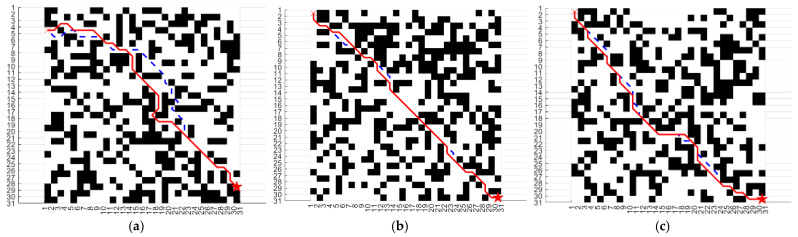
This is the experiment of simulation environment. Red lines mean the results of improved algorithm, blue dotted lines mean the results of traditional algorithm, red stars mean the results of goal nodes: (**a**) grid 1; (**b**) grid 2; (**c**) grid 3.

Three different 31 × 31 grid maps were generated, and path planning was performed on each map using both the traditional A* algorithm (indicated by dashed lines) and the A* algorithm enhanced with a modified heuristic function (indicated by solid lines), as illustrated in Figure 1.

As shown in Table 1, with the improved heuristic function, while maintaining the same number of path nodes, the number of nodes traversed was reduced by 22.78%, 9.93%, and 13.49%, respectively; the total cost was reduced by 22.89%, 8.41%, and 8.90%, respectively; the number of turning points was reduced by 11.26%, 12.96%, and 19.85%, respectively; and the running time was reduced by 20.49%, 27.60%, and 28.04%, respectively. These improvements in the A* algorithm’s computational metrics significantly enhance its operational efficiency and speed.

#### 2.2.2. Improved Neighborhood Search Strategy

**Figure 2:**
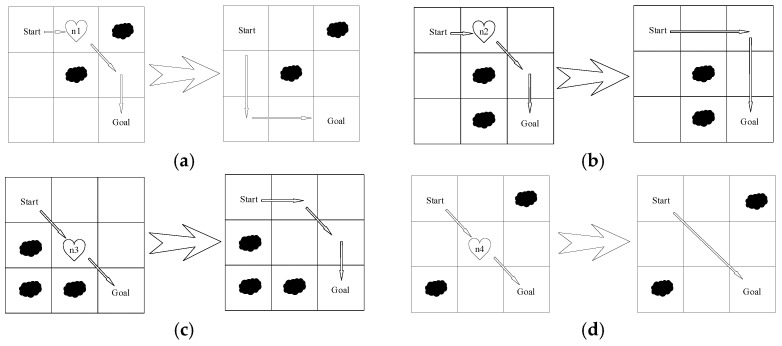
The process of optimized method: (**a**) Node 1 optimized method; (**b**) Node 2 optimized method; (**c**) Node 3 optimized method; (**d**) Node 4 optimized method.

The traditional A* algorithm uses an 8-neighbor search strategy, where the search begins at the central node and the surrounding 8 adjacent grid nodes are considered as potential moves. The optimal child node is selected for expansion based on the evaluation function’s results. However, without accounting for obstacles, the mobile robot might traverse obstacles diagonally, leading to collisions and other issues. To address this, a child node selection strategy is introduced, with its key step being the evaluation of the relative positional relationship between child nodes and obstacles. Based on the evaluation results, unsafe child nodes can be effectively excluded, thus avoiding collisions. The evaluation process is outlined as follows:

1. As shown in Figure 2a,c, let the subnode be $\left(x,y\right)$. If the obstacle is located at $\left(x,y+1\right)$ or $\left(x,y-1\right)$, then $\left(-1,1\right)$, $\left(0,1\right)$, $\left(1,1\right)$ or $\left(-1,-1\right)$, $\left(0,-1\right)$, $\left(1,-1\right)$ are removed;
2. As shown in Figure 2b, let the subnode be $\left(x-1,y\right)$ or $\left(x+1,y\right)$, then $\left(-1,1\right)$, $\left(-1,0\right)$, $\left(-1,-1\right)$ or $\left(1,1\right)$, $\left(1,0\right)$, $\left(1,-1\right)$ are removed;
3. As shown in Figure 2d, if the obstacle is located on opposite sides of the diagonal of the child node, it is not necessary to remove the child node.

#### 2.2.3. Smoothing Curve

The traditional A* algorithm often generates an excessive number of irrelevant turning points during path planning, resulting in paths with numerous sharp segments. Such paths can cause rapid changes in speed and acceleration for the vehicle, particularly at turns, which increases the risk of sharp turning, tipping over, or colliding with obstacles. To address this issue, a method utilizing cubic Bezier curves for smoothing and optimization has been proposed [23]. The optimized path is smoother, enhancing both the safety and feasibility of the trajectory. The formula for the cubic Bezier curve is as follows:

(9) $$B(t)=P_{0}{\left(1-t\right)}^{3}++3P_{1}t{\left(1-t\right)}^{2}+3P_{2}t^{2}(1-t)+P_{3}t^{3},t\in [0,1]$$

Suppose there are two path points $P_{0}$ and $P_{1}$, with coordinates $\left(x_{0},y_{0}\right)$ and $\left(x_{1},y_{1}\right)$, and orientations $\theta_{0}$ and $\theta_{1}$. $\left(P_{0},P_{1},P_{2},P_{3}\right)$ are the four control points of the Bezier curve. The curve starts at $P_{0}$, $P_{2}$ and moves toward $P_{1}$, $P_{3}$. The specific formula for the cubic Bezier curve is as follows:

(10) $$\left\{\begin{array}{c} P_{0}=P_{start}\\ P_{1}=P_{start}+\frac{\sqrt{{\left(x_{0}-x_{1}\right)}^{2}+{\left(y_{0}-y_{1}\right)}^{2}}}{4}\times \left(\cos\theta_{0},\sin\theta_{0}\right)\\ P_{2}=P_{goal}-\frac{\sqrt{{\left(x_{0}-x_{1}\right)}^{2}+{\left(y_{0}-y_{1}\right)}^{2}}}{4}\times \left(\cos\theta_{1},\sin\theta_{1}\right)\\ P_{3}=P_{goal} \end{array}\right.$$

A cubic Bezier curve generates an optimized smooth path by calculating each segment individually and then combining them. When multiple control points are present along the path, the cubic Bezier curve effectively avoids filtering out critical nodes during the optimization process, ensuring the continuity of the path and achieving the optimal solution.

#### 2.2.4. Result

**Figure 3:**
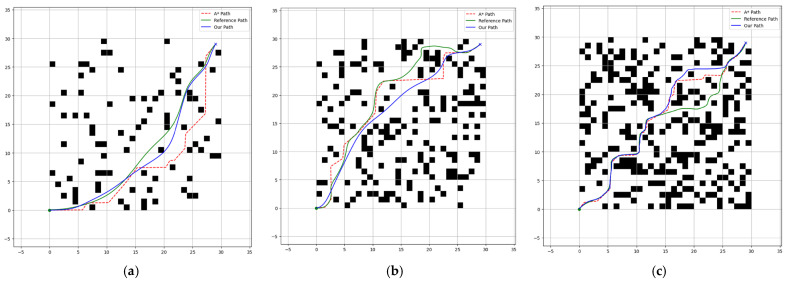
Grid map: (**a**) 10% obstacles; (**b**) 20% obstacles; (**c**) 30% obstacles.

To visually demonstrate the improvements in the heuristic function, neighborhood search strategy, and curve smoothing of the traditional A* algorithm, this section employs Matplotlib (version 3.10.1) to visualize the path planning results. Three simulation experiments were conducted on 30 × 30 grid maps with obstacle densities of 10%, 20%, and 30%, as shown in Figure 3a–c. In these figures, the green solid circle and blue cross represent the start and end points, respectively; the red dashed line indicates the path generated by the original A* algorithm; the green solid line represents the improved A* algorithm from the reference [24]—using 4-neighborhood search and second-order Bézier curve smoothing; and the blue solid line shows our improved A* algorithm. Black squares denote obstacles sized 1 × 1.

With the starting point, ending point, and global obstacle positions held constant, the parameter comparison results are summarized in Table 2.

The experiment averages the simulation results from grid maps with obstacle densities of 10%, 20%, and 30%. Compared with the parameter data output by the traditional A* algorithm and the reference algorithm, the average path length of the improved algorithm was reduced by 10.73% and 7.34%, respectively; the running time was reduced by 19.70% and 11.42%, respectively; the number of search nodes was reduced by 12.80% and 8.80%, respectively; the total cost was reduced by 16.57% and 9.72%, respectively; and the number of turning points was reduced by 17.75% and 11.67%, respectively.

The traditional A* algorithm tends to generate paths with multiple turning points to achieve the shortest route, which often leads to frequent turns near obstacles, lacking smoothness and stability. Although the path length might not be overly long, such paths fail to meet the ideal criteria for optimality. In contrast, our improved algorithm significantly reduces right-angle turns, shortens the path length, and effectively lowers the operational cost by optimizing both the neighborhood search and curve smoothing. This results in a path that is not only shorter but also smoother, with fewer turning points and reduced overall cost—thereby markedly enhancing system stability. The parameter outputs in Table 2 further confirm the feasibility, effectiveness, and superiority of the improved algorithm in global path planning.

### 2.3. Hybrid Algorithm

After implementing the aforementioned optimization strategies, the improved A* algorithm is capable of providing an optimal solution for global path planning, making it well-suited for finding the best path in static working environments. However, when an unknown obstacle suddenly appears in the mapped environment, the algorithm struggles to perform dynamic obstacle avoidance and local path planning.

**Figure 4:**
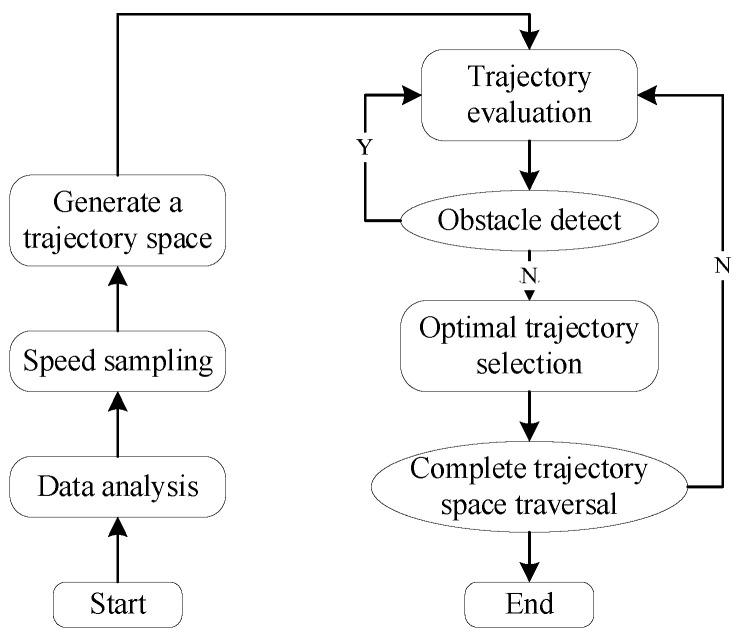
Step-by-step process of the DWA algorithm.

The Dynamic Window Approach is a velocity-sampling-based local path planning method that optimizes the path by constraining velocity and finding the optimal solution within the velocity space to achieve dynamic obstacle avoidance. The diagram shown in Figure 4 illustrates the step-by-step process of the DWA algorithm. First, the velocity model of the robot within its motion space is collected and constructed, and initialization parameters are set, including the start point, end point, obstacles, velocity, and acceleration. Then, based on the current state and parameters of the robot, the algorithm computes the state for the next short time interval. Each trajectory is evaluated according to a scoring standard, and the trajectory with the highest score is selected as the safe route for obstacle avoidance during navigation. This selected trajectory serves as the basis for autonomous navigation within the specified area. At the same time, an evaluation function is used to assess the path and select the optimal solution, with the robot’s state and position being continuously updated until the destination is reached. The DWA algorithm function is called by the system to assist the robot in dynamic obstacle avoidance in a simulated environment, enabling it to effectively respond to obstacles in dynamic conditions while ensuring safety and efficiency.

In local path planning, the current position and velocity are updated, as indicated by Equation (11). The kinematic model is constructed to sample the robot’s linear υ and angular velocities ω, thereby describing its motion trajectory. At each time step △t, the robot continuously updates its state based on the current linear and angular velocities, adjusting its position $\left(x,y\right)$ and speed θ to achieve both obstacle avoidance and path planning in a dynamic environment. The dynamic obstacle avoidance problem is transformed into three constraints within the velocity space.

(11) $$\left\{\begin{array}{l} x=x+\upsilon_{x}△t\cos\theta_{t}-\upsilon_{y}△t\sin\theta_{t}\\ y=y+\upsilon_{x}△t\sin\theta_{t}+\upsilon_{y}△t\cos\theta_{t}\\ \theta_{t}=\theta_{t}+\omega_{t}△t \end{array}\right.$$

To optimize the aforementioned constraints, the DWA algorithm introduces an evaluation function J to optimize the motion trajectory by comprehensively considering the forward speed, obstacle avoidance performance, and dynamic constraints, while scoring each candidate trajectory. The evaluation function J is defined as shown in the following Equation (12).

(12) $$J(\upsilon ,\omega )=\alpha \cdot J_{forward}+\beta \cdot J_{obstacle}+\gamma \cdot J_{dynamic}$$

**Figure 5:**
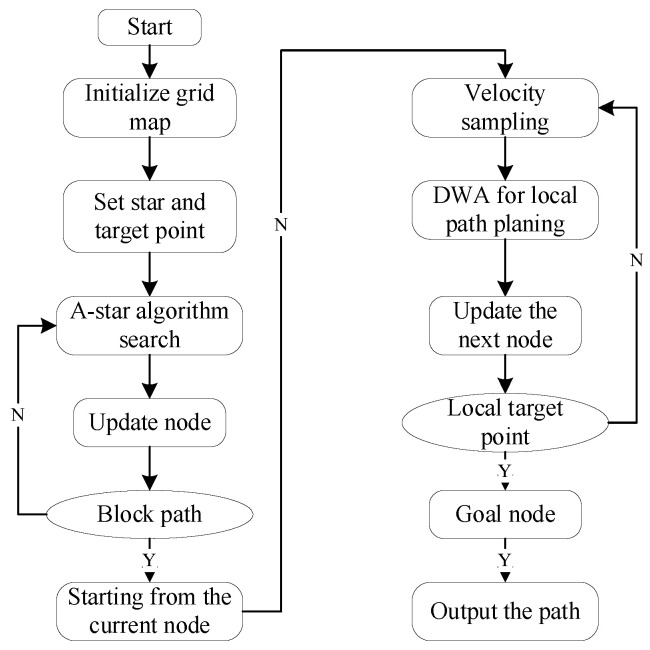
The chart of fusion algorithm.

The coefficient α,β,γ is a weighting function that adjusts the importance of each factor through weighted summation; the evaluation function $J_{forward}$ measures the robot’s forward speed toward the target; the evaluation function $J_{obstacle}$ quantifies the minimum distance between the trajectory and obstacles, ensuring effective obstacle avoidance; and the evaluation function $J_{dynamic}$ assesses whether the robot’s dynamic constraints are satisfied, such as the limits on maximum speed and maximum acceleration. The key global path points generated by the A* algorithm can be incorporated into the DWA algorithm as guiding points for local path planning. This integration ensures a balance between global optimality and local obstacle avoidance capability [25]. The flowchart of the hybrid algorithm is shown in Figure 5, illustrating the entire process from global path planning to local obstacle avoidance.

The fusion steps are as follows. When using an improved A* algorithm for path planning, an optimal path is generated based on the current starting point and target. By refining the heuristic function, the algorithm reduces unnecessary backtracking and avoids redundant turning points. When the robot reaches a node in the target area, the current node’s position is updated as the new starting point, and the target can be reset to the coordinates of the next target area. Each time the robot reaches a new target node, the system automatically replans the path by repeatedly invoking the improved A* algorithm to continually update the starting point and target, ensuring that the robot always moves along the optimal path until the entire predetermined route is completed. If unknown obstacles or dynamic obstacles requiring avoidance are encountered, the robot will utilize the DWA algorithm for local obstacle avoidance. This local avoidance behavior is achieved by continuously evaluating the surrounding environment and adjusting the robot’s trajectory to avoid collisions, while the system simultaneously maintains the global optimality of the path. Through these steps, the robot’s stability and efficiency in dynamic environments are ensured.

### 2.4. Processing of Sensor Noise

Kalman filtering is an autoregressive filtering method based on the minimum variance criterion and is fundamentally rooted in Bayesian filtering theory. The basic mathematical model can be expressed as follows:

(13) $$\left\{\begin{array}{l} x_{t}=ax_{t-1}+bi_{t}+c\\ y_{t}=hx_{t}+v_{t} \end{array}\right.$$

where *x* represents the robot’s state over time in the environment, *a* is the state transition matrix, *b* is the control input matrix, *c* stands for the prior estimate, *y* is the observation value provided by the robot’s sensors, *h* is the observation matrix, and *v* denotes the measurement noise.

In our path planning algorithm, various improvements have been implemented to achieve superior performance compared to other algorithms. However, during validation, noise interference from devices such as lidar (e.g., Gaussian noise) was not initially considered. This noise can lead to unnecessary oscillations and pose adjustments, resulting in redundant waypoints along the planned path. To address this, Kalman filtering is introduced to smooth out noisy data. The key idea is to filter out nodes with large step sizes using the Kalman gain. The robot uses a known dynamic model to predict the next state and its error covariance, then calculates an adjusted Kalman gain based on the predicted covariance and the noise measurements. This process allows the Kalman filter to provide a more accurate estimation for nodes with significant step sizes until further search via that node becomes unfeasible. The Kalman gain at the *n* iteration is given as follows:

(14) $$K_{n}=\frac{p_{n}h^{T}}{hp_{n}h^{T}+r}$$

where *p* is the error covariance matrix of the nodes and *r* is the covariance matrix representing the measurement noise of the path nodes.

**Figure 6:**
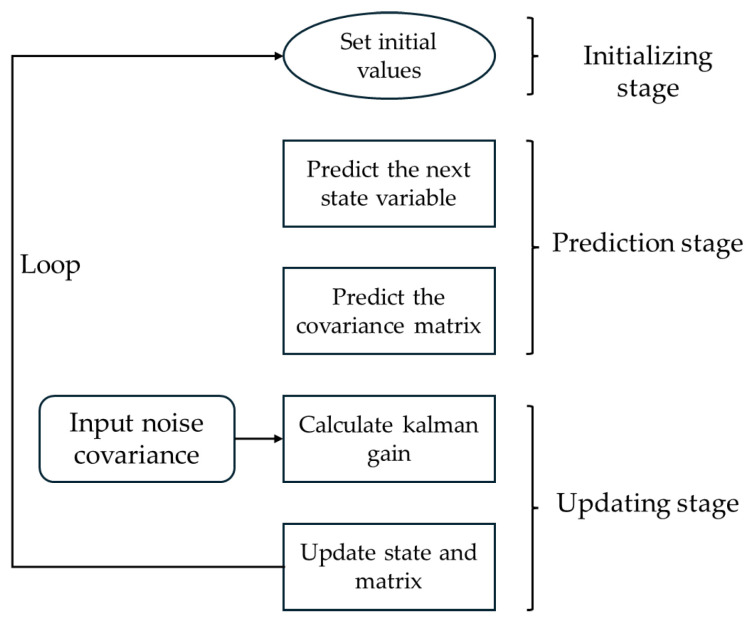
The process flow of Kalman filtering.

In the context of path planning, eliminating redundant points leads to a more concise path, thereby improving search efficiency. The process flow based on the principles of the Kalman filter is illustrated in Figure 6. This flow involves mapping through the observation matrix to estimate node states, predicting the future positions of nodes, and evaluating the necessity of each node. Consequently, the Kalman filter helps improve the path planning performance of the algorithm in noisy environments.

## 3. Experiment Verification

To verify the effectiveness of the fusion algorithm for path planning, this section discusses the experimental procedures in both simulation and real environments in detail. Section 3.1 presents the specific structure of the mobile robot. Section 3.2 provides a brief introduction to SLAM algorithms, with the Cartographer algorithm being selected. Section 3.3 focuses on simulation experiments: a ROS platform is set up, the structure of the AMCL localization algorithm is introduced, and Gazebo (version 11.11.0) and RViz (version 1.14.25) software are used for joint simulation. In RViz, the local cost map generated when the robot employs the DWA algorithm is displayed, and finally, the fusion algorithm is applied to complete the path planning, generating an optimal path. Section 3.4 discusses the performance of the fusion algorithm in real-world experiments, where the robot demonstrates excellent localization and obstacle avoidance capabilities.

### 3.1. The Structure of Robot

**Figure 7:**
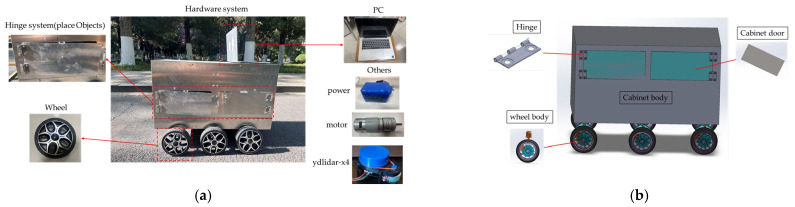
The diagram of the robot: (**a**) Hardware System; (**b**) 3D model of SolidWorks.

The mobile robot used in this experiment is a self-developed six-wheeled robot, the schematic diagram of the components of the hardware system is shown in Figure 7a. The robot features a design combining a box-shaped body with omnidirectional wheels, equipped with an integrated suspension mechanism and a six-wheel chassis system. It is outfitted with a PC running a ROS system and a LiDAR sensor to generate 2D maps of the surrounding environment and collect real-time motion data, providing hardware support for path planning. The point cloud data generated by the LiDAR are processed to create a cost map, where different regions are assigned varying cost values to represent the traversal cost in path planning [26]. The cost map is stored in a grid format, with obstacles in the environment marked as high-cost areas, indicating regions the robot must avoid. The cost map is updated in real time based on data from the LiDAR and other sensor inputs, significantly enhancing the robot’s ability to perceive dynamic environments [27]. This capability provides robust support for autonomous navigation. A wheel-based damping module is installed to reduce vibrations and errors during movement between the chassis and the drive motors. Once the system is operational, the robot can build a 2D map and adjust its motion via the drive motors to follow the planned path. Figure 7b shows the 3D assembled robot model, which was converted into a URDF file using the URDF plugin. The model was subsequently imported into the ROS system for control and simulation experiments.

### 3.2. The Selection of Slam Algorithms

In robot path planning, common 2D LiDAR SLAM methods include the following: Hector, based on scan matching; Gmapping, based on particle filtering; and Cartographer, based on graph optimization [28].

Cartographer introduces several optimizations for loop closure and map refinement. First, it employs a branch-and-bound method to accelerate search speed, using matched sub-maps as constraints to reduce redundant computations. Next, it optimizes the pose graph through loop closure, reducing uncertainties and refining trajectory estimates. Finally, it applies global map optimization, enabling local map updates and providing more precise solutions for map building and robot localization. These improvements make the system highly stable when adapting to environmental changes.

Th Cartographer algorithm, renowned for its flexibility and robustness in SLAM applications, employs loop closure detection and back-end optimization to correct cumulative errors. By integrating submap matching with global optimization, it significantly enhances both real-time performance and accuracy. Additionally, its fusion of multiple sensor inputs bolsters the stability of localization and mapping, ultimately elevating the robot’s ability to construct precise 2D models. Consequently, opting for Cartographer in robotic simulation experiments for environmental mapping represents the most optimal choice.

### 3.3. Simulation Experiments

In the simulation experiments, this study utilized the ROS Noetic system, with pre-installed tools, including Gazebo and RViz. Gazebo provides a simulated environment that facilitates mobile robot motion simulation and algorithm optimization, while RViz serves as a visualization tool for ROS data, enabling users to intuitively observe sensor data, robot status, and environmental perception results [29]. The ROS platform is characterized by its high portability, modular structure, and ability to handle messages in parallel [30]. Its modular architecture allows for customized message transmission, with nodes subscribing to and publishing topics as needed.

The simulation experiments were conducted on a PC using ROS and Gazebo. Table 3 outlines the configuration of the 2D LiDAR SLAM platform.

**Figure 8:**
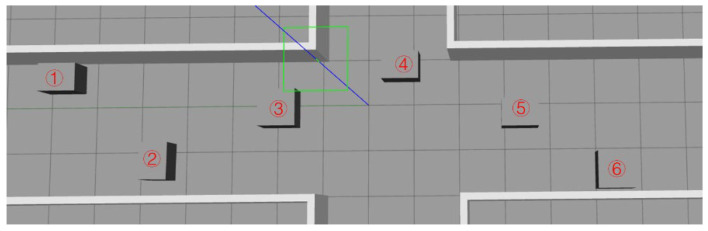
This is the gazebo world of the simulated environment. The green box and blue line represent the central coordinate of the environmental map, the numbers represent the serial numbers of the six obstacles.

In the Gazebo simulation world, an indoor corridor is constructed with dimensions of 18 × 3 × 2 units (length, width, and height, respectively) and an ideally smooth terrain, as depicted in Figure 8. Along the corridor, six rectangular obstacles are positioned at the following coordinates: obstacle 1 at (0.6, 6.7), obstacle 2 at (−1.2, 4.5), obstacle 3 at (−0.1, 1.9), obstacle 4 at (0.8, −0.7), obstacle 5 at (−0.2, −3.3), and obstacle 6 at (−1.5, −5.4). To introduce randomness in obstacle sizes, obstacles 1, 2, 4, and 5 have dimensions of (0.6, 0.8, 1), while obstacles 3 and 6 measure (0.8, 0.8, 1). These obstacles are arranged in a sine curve pattern, ensuring that there is a minimum distance of 1 unit between each obstacle.

**Figure 9:**
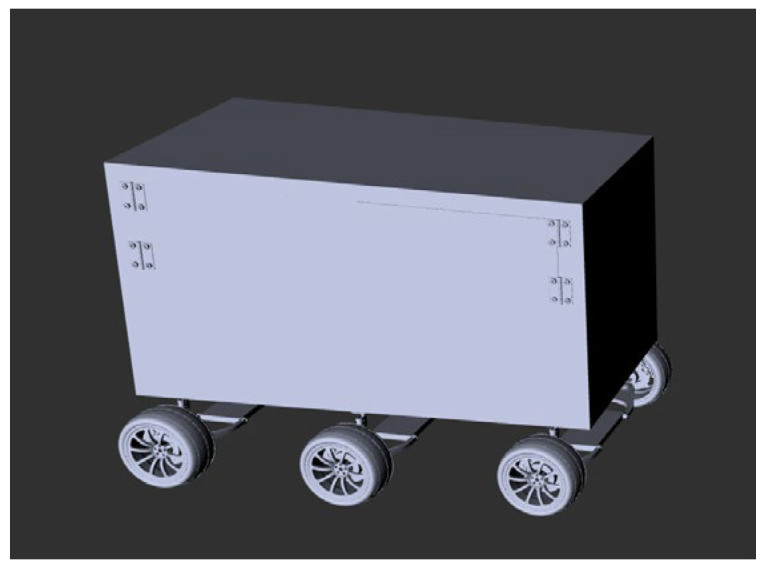
Mobile robot URDF model simulation.

During the experiments, the mobile robot dynamically acquired data from the simulated corridor and sidewalk, avoided pre-placed obstacles, and followed a predefined path to reach target points [31]. The simulation model of the mobile robot and LiDAR was created using a URDF file, as shown in Figure 9.

In this experiment, a simulation environment for the operation of the mobile robot was constructed in Gazebo software. The current state parameters of the robot were obtained using sensors such as LiDAR and IMU. LiDAR sensors utilize point cloud processing algorithms to extract detailed environmental features and obstacle information. Although they capture fine environmental details, their lower update frequency still enables effective path planning and obstacle detection. In contrast, IMU sensors collect vehicle speed data and estimate the vehicle’s pose and motion state at a high update rate, though they are susceptible to drift. To fully leverage the strengths of these diverse sensors, data fusion is typically employed to mitigate their respective limitations. In our approach, Kalman filtering is used for sensor fusion, significantly improving robustness and yielding more precise pose and velocity estimates, particularly in complex, dynamic environments. However, the considerable difference in sampling frequencies between LiDAR and IMU results in mismatched timestamps. To overcome this, we make use of the ROS package “message_filters”, which aligns data based on the nearest timestamps. Additionally, the integration of multiple sensors can lead to increased computational complexity, potentially compromising real-time performance. In such scenarios, employing GPU or FPGA acceleration for point cloud processing, adjusting simulation step sizes, and optimizing resource allocation can help maintain efficient operation.

The ROS system published odometry information [32], subscribed to velocity commands, and executed path planning tasks through the chassis control module while providing real-time feedback on the robot’s motion state, thereby achieving autonomous navigation. During the simulation experiment, the Cartographer algorithm was selected for 2D grid map construction. The results were saved in image format and visualized using RViz. Through the Navigation2 Goal feature, navigation target points were set in the simulation environment, enabling autonomous navigation and map building. The LiDAR determined the position and distance of obstacles through beam ranging, generating point cloud data of the surrounding environment [33] and updating the 2D map in real time.

**Figure 10:**
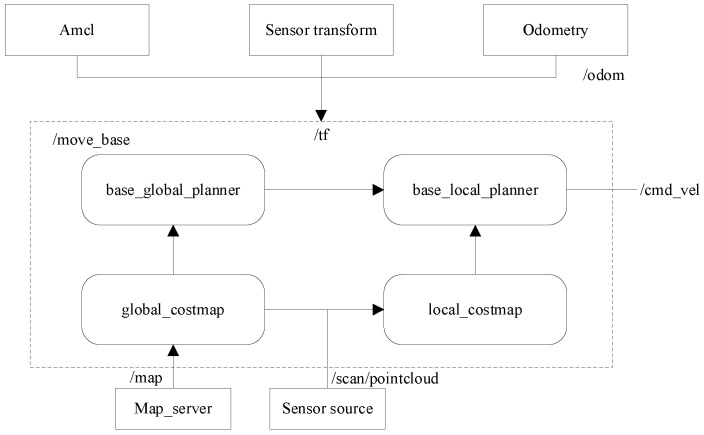
Schematic of navigation process.

Additionally, by selecting the start and end points of the path on the map, the algorithms before and after improvement could be compared visually. The move_base module in the ROS framework handled the parameters for various aspects of path planning, integrating Global Costmap and Local Costmap, and subscribing to multiple sensor inputs, including AMCL localization and LiDAR data. A schematic of the navigation process is shown in Figure 10. The underlying control system encapsulated and published velocity command topics (cmd_vel) through compiled programs. The current target point was stored in “move_base_simple_goal”. During the robot’s movement, the robot’s coordinates and timestamps were recorded by subscribing to position topics. Operational commands were sent to the STM32 via serial communication, driving the robot to navigate to the target.

**Figure 11:**
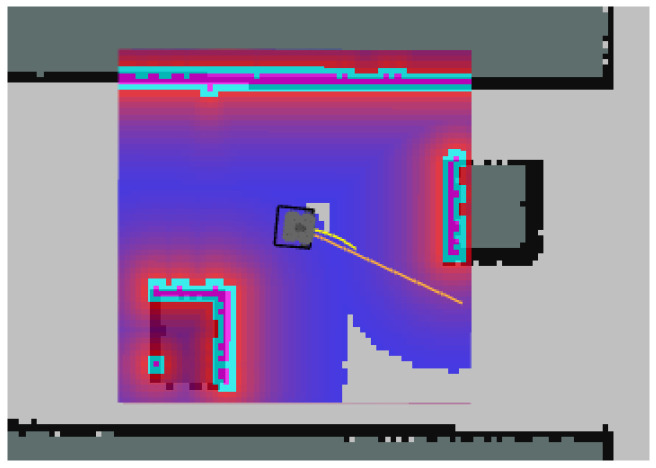
Local costmap for DWA algorithm. The dark blue and red block area represents the maximum scanning range covered by the odometry; the cyan line segment represents the boundary of the detected obstacle.

The local costmap is a real-time generated local map based on sensor data such as LiDAR and cameras, used to reflect the distribution of obstacles around the robot. In the simulation environment, the dark blue block area represents the maximum scanning range covered by the odometry, which dynamically updates as the robot moves. The orange lines represent the globally optimal path generated by the DWA algorithm in the local planner, while the yellow lines indicate the locally optimal path generated by the local planner. A schematic of the local costmap is shown in Figure 11.

**Figure 12:**
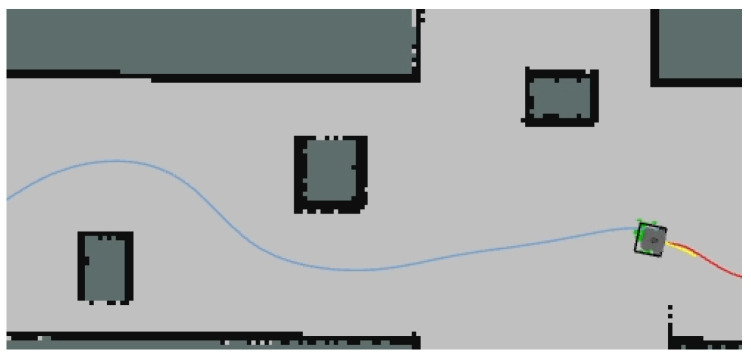
Optimal roadmap. the gray area represents the robot’s traversable region; the black thick lines represent the obstacle edges; the red line represents the globally optimized path planned; the blue line shows the trajectory already traveled by the robot.

The turning points in the robot’s movement path are typically locations where a significant change in direction occurs. These turning points can be identified by calculating the directional vector of the robot’s position changes. If the directional difference between two consecutive positions exceeds a certain threshold, the point is considered a turning point. Additionally, if the positional change distance of the robot exceeds a specified threshold, the point can also be treated as a turning point. Upon detecting a new turning point, a counter can be used to track the number of turning points, and the corresponding timestamps can be recorded to calculate the travel time between adjacent turning points. As shown in Figure 12, the gray area represents the robot’s traversable region, while the black lines indicate the obstacle edges detected by sensors. The red line represents the globally optimized path planned, and the blue line shows the trajectory already traveled by the robot. This provides a visual representation of the results after integrating the optimization algorithm, which is displayed in RViz by subscribing to the robot path node.

**Figure 13:**
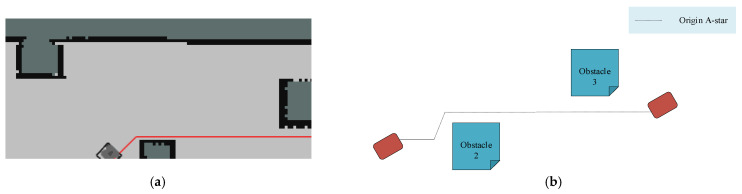

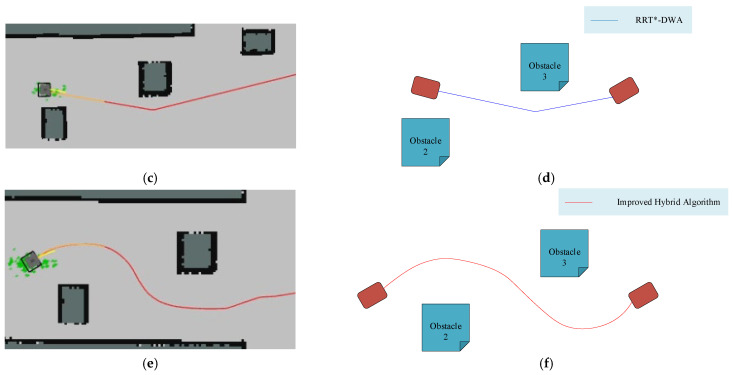
Comparison experiments of path planning: (**a**) Simulation experiment of A* algorithm; (**b**) Schematic diagram of A* algorithm; (**c**) Simulation experiment of RRT*-DWA fusion algorithm; (**d**) Schematic diagram of RRT*-DWA fusion algorithm; (**e**) Simulation experiment of improved fusion algorithm; (**f**) Schematic diagrams of improved fusion algorithm. In (**a**,**c**,**e**), the red color line represents the global path; the yellow line represents the local route in the local path planning; the orange line represents the global route in the local path planning.

The core of implementing the local path planning functionality lies in dynamically adjusting the path to cope with interference from local obstacles. When the robot detects new obstacles, the current path is corrected, and a feasible path is regenerated. In this experiment, the local planner divides the planning process into multiple time modules. Based on the dynamic constraints of the robot and obstacle constraints, it generates a set of trajectory paths that satisfy the conditions. The improved path planning method generates paths that meet nonholonomic constraints by considering the kinematic characteristics of the robot. Figure 13 shows the results of the path planning comparison experiments. By comparing the improved fusion algorithm proposed in this paper with the traditional algorithm and the RRT*-DWA fusion algorithm, the superiority of our approach in path planning is demonstrated more intuitively. Specifically, Figure 13a,c,e displays the paths generated by the traditional A* algorithm, the RRT*-DWA fusion algorithm, and our improved fusion algorithm, respectively; Figure 13b,d,f presents schematic diagrams of the corresponding paths when encountering obstacles 2 and 3. Comparative analysis indicates that the traditional A* algorithm tends to overlook the minimum safety distance between the robot and obstacles during path planning, increasing the risk of collision. In practical operation, the robot may enter critical areas near obstacles where it fails to find an optimal path, forcing it to backtrack and re-search. Furthermore, when new obstacles appear in the path, the robot may rotate in place and enter a delayed decision-making state, failing to respond accurately in time. This leads to reduced speed and delayed turning maneuvers.

The improved algorithm ensures a safe distance between the robot and obstacles through reasonable optimization in global path planning. It also smooths the path to minimize turning costs while keeping the path length minimal. The optimization results demonstrate that as the path length increases, the number and complexity of turns significantly decrease, effectively enhancing search efficiency. This results in more efficient autonomous navigation and ensures stable operation of the robot in complex environments.

### 3.4. Real Experiments

**Figure 14:**
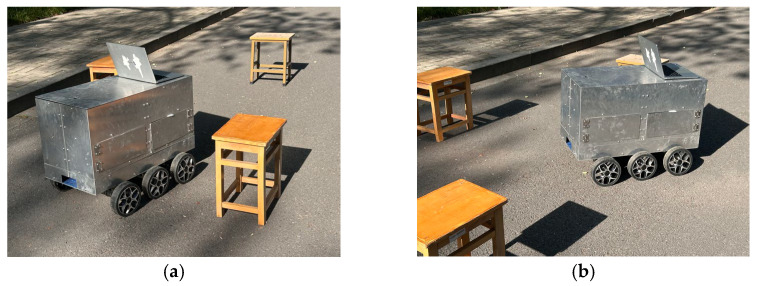
Experimental process: (**a**) state 1; (**b**) state 2.

For the outdoor experiment, the robot’s chassis system ran on the Ubuntu 20.04 operating system with Noetic-ROS and was equipped with a 2D LiDAR module to implement 2D laser SLAM mapping using the Cartographer algorithm. In the Noetic-ROS interface, navigation target points were set using the 2D Nav Goal feature in RViz. The experimental scenario simulated the actual environment of the road intersection near the Linji Building at the Xinzhuang Campus of Nanjing Forestry University. Commands were sent from the upper computer to enable the robot to autonomously complete on-site testing. In the field test, the robot encountered three obstacles sequentially and adjusted its orientation and pose during navigation to avoid them, as shown in Figure 14a,b.

**Figure 15:**
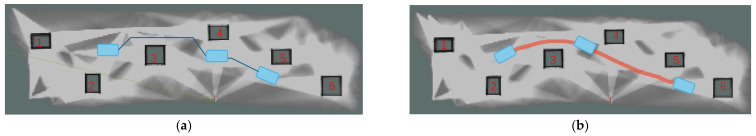
The figures represent SLAM mapping results; the numbers represent the serial numbers of the six obstacles: (**a**) Original A* algorithm; (**b**) Improved A* and DWA algorithm.

During the test, the LiDAR collected environmental data to construct the map. The 2D laser SLAM environment and mapping results are shown in Figure 15. The robot system successfully built an environmental map of the intersection area. The visualized trajectory diagrams of the robot’s navigation routes using the traditional A* algorithm and the improved algorithm are shown in Figure 15a,b, respectively. The system achieved a mapping and localization accuracy of approximately 0.7 m, with a stable operational frame rate of 26–30 FPS. Although mapping and navigation in outdoor environments introduced some errors and delays compared to indoor settings, these issues had minimal impact on the mapping results for short-path generation.

The output data of various comparative parameters during the robot’s navigation are summarized in Table 4.

Experimental results demonstrate that, compared to the A* and DWA algorithms, the improved hybrid algorithm enhances search efficiency by 10.93%, reduces search node count by 32.26%, decreases the number of turning points by 36.36% and the value of turning angle by 34.83%, shortens the total path length by 22.05%, and improves overall path smoothness. The proposed hybrid algorithm is more stable, significantly reduces collision probability, and demonstrates its applicability for mobile robot localization and navigation in real-world environments.

**Figure 16:**
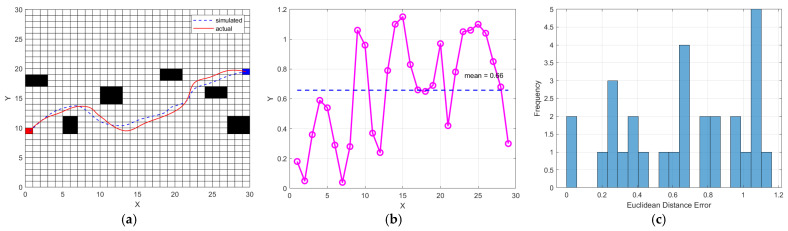
Analysis graphs: (**a**) Trajectory comparison; (**b**) Error analysis, red cell represents the euclidean distance error of path points; (**c**) Error distribution, blue cell represents the occurrence frequency of the euclidean distance error.

The collected actual trajectory coordinates and simulated path coordinates are integrated and imported into the MATLAB(R2024a) simulation tool, including the position coordinates. The coordinate step was set to one. The resulting trajectory comparison plot is shown in Figure 16a below. In the plot, the X-axis represents the robot’s lateral coordinates, and the Y-axis represents the longitudinal coordinates. The blue dashed line indicates the simulated path, while the red solid line shows the actual trajectory, providing a clear visualization of the path deviation trend and serving as a basis for preliminary comparative analysis. The error is calculated using the Euclidean distance formula, as shown below:

(15) $$EDE\left(x\right)=\sqrt{{\left(x_{actual}-x_{simulated}\right)}^{2}+{\left(y_{actual}-y_{simulated}\right)}^{2}}$$

The Euclidean Distance Error $EDE\left(x\right)$ represents the deviation between the simulated path and the actual trajectory. Ideally, the error should be zero, meaning that the simulated path perfectly matches the actual trajectory. However, this is often difficult to achieve in practice, so the goal is to minimize the error. A smaller error indicates higher accuracy in both path planning and trajectory tracking, ensuring that the robot follows the intended route precisely without deviating from the predetermined path.

Figure 16b illustrates the error analysis, where the X-axis represents the coordinate step and the Y-axis represents the Euclidean Distance Error. The average error is calculated to be 0.66. The plot visually identifies locations where the error significantly increases, which may be attributed to factors such as turning or encountering obstacles.

Figure 16c displays the error distribution, with the X-axis representing the Euclidean Distance Error and the Y-axis indicating the frequency of occurrence. This distribution helps in understanding the concentration of errors and identifying any outliers, enabling a rapid assessment of the overall error trend.

## 4. Discussion

**Figure 17:**
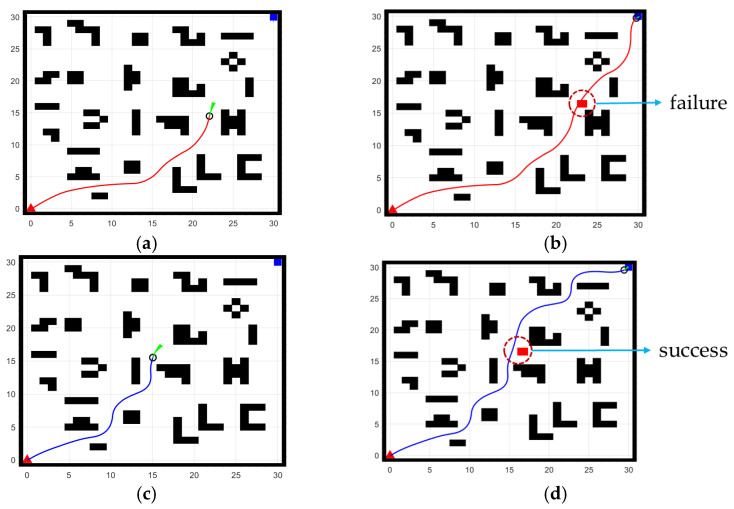
Path planning routes, red solid line denotes the trajectory produced by the reference algorithm, blue solid line represents the trajectory produced by our algorithm: (**a**) Search result of the reference algorithm; (**b**) Full path of the reference algorithm; (**c**) Search result of our algorithm; (**d**) Full path of our algorithm.

To verify the effectiveness of the proposed algorithm in a highly dynamic environment, where obstacles change rapidly, and to discuss its characteristics and advantages compared to existing research algorithms, experiments were conducted using a reference algorithm [34] and our improved algorithm. The experimental environment was a 30 × 30 grid with multiple obstacles distributed throughout, as shown in Figure 17. In the figure, the red upward-pointing triangle indicates the starting point, the blue square represents the target point, and the green fan-shaped area shows the region being searched by the robot. The red solid line denotes the trajectory produced by the reference algorithm, while the blue solid line represents the trajectory produced by our algorithm. Figure 17a,c illustrates the search states during path planning for the reference algorithm and our algorithm, respectively. In these search processes, the planning strategies are different, with each algorithm proceeding along its respective search direction. Figure 17b,d depicts the complete search paths generated by the two algorithms.

During the robot’s operation, a red square obstacle was dynamically introduced. As shown in Figure 17b,d, the reference algorithm failed to completely avoid the obstacle in time, with the trajectory partially intersecting the dynamic obstacle. In contrast, our improved algorithm successfully avoided the obstacle and maintained movement along the predetermined path. This outcome verifies the effectiveness of the improved algorithm in dynamic environments. Table 5 below summarizes the parameter values output by the two algorithms during path planning.

As shown in Table 5, our algorithm achieves a trajectory length reduction of approximately 22.05% compared to the reference algorithm, a reduction in turning angle by 16.85%, a decrease in the number of turning points by about 27.79%, and a reduction in search time by 13.98%. These comparative results indicate that our fusion algorithm can more stably find the global optimal path, reducing the probability of re-searching or deviating from the optimal route. Moreover, during operation, it quickly plans an appropriate path, thereby minimizing the chance of errors.

In parks or mountainous regions where slopes are steep or the terrain is uneven, mobile robots can adaptively head toward their targets; however, the performance of sensor fusion algorithms may deteriorate or even fail, thus limiting their full potential. In such complex environments, issues including sensor noise, environmental fluctuations, high-resolution computational costs, and overall time expenditures can compromise the effectiveness of global path planning.

In simple 2D environments, the improved algorithm presented in this paper demonstrates good application performance and value. However, in three-dimensional environments with more obstacles, varying height levels, and irregular layouts, several issues arise, including reduced path smoothness, increased collision rates, diminished path safety, and higher energy consumption. Therefore, in order to address these challenges, our future work on this fusion algorithm will need to focus on enhancing the performance of various parameters in 3D environments. To address the array of issues arising from three-dimensional environments in our experiments, we plan to replace the current scanning range of the lidar device with a range of 3D lidar devices in future work. We will also further optimize our current fusion path planning algorithm to better adapt to complex and irregular 3D environments.

Future research will employ a dual-layer mapping technique, combining hierarchical global and local planning to adjust the path in real time. Alternatively, incorporating machine learning methods, such as deep learning, allows for dynamic adjustments to the heuristic estimation function based on historical data and current conditions, thereby improving both search efficiency and path quality. These strategies offer promising avenues for the future development of path planning algorithms.

## 5. Conclusions

To address the issues of excessive redundant nodes, lack of smoothness in the path, and low search efficiency in the traditional A* algorithm, this paper proposes an improved A* algorithm for global path planning. The improved algorithm introduces and adjusts an exponential weight coefficient in the heuristic function $h\left(n\right)$, reconstructing the dynamic weight function $\omega \left(n\right)$ to enhance the evaluation function. This effectively reduces the number of expanded nodes and the search time. Additionally, the algorithm optimizes the search neighborhood by using the azimuth angles of the start and target nodes to directionally filter the search neighborhood. It replaces the traditional 8-neighborhood search with a 5-neighborhood search, significantly reducing the algorithm’s runtime and the number of path nodes to evaluate. Furthermore, the algorithm smooths the path using segmented Bezier curves, which reduces curvature and path length, thereby improving the smoothness and efficiency of the path. This ensures the robot reaches the target location safely, smoothly, and quickly with minimal turning costs. For local path planning, the Dynamic Window Approach is employed to generate the optimal path, while the improved A* algorithm provides multiple waypoints as sub-goals for the DWA algorithm. By searching and predicting within the local space, the final optimal path is generated.

In Section 2.2.4, path planning experiments were conducted on grid maps with obstacle densities of 10%, 20%, and 30%. The improved algorithm was compared against the unmodified A* algorithm, as well as the algorithm in reference [24]. The average path length of the improved algorithm was reduced by 10.73% and 7.34%, respectively; the running time was reduced by 19.70% and 11.42%, respectively; the number of search nodes was reduced by 12.80% and 8.80%, respectively; the total cost was reduced by 16.57% and 9.72%, respectively; the number of turning points was reduced by 17.75% and 11.67%, respectively. All three methods were evaluated in environments with different obstacle densities. The comparative results demonstrate that the parameters for the improved algorithm show a consistent optimization trend over both the original A* and the reference algorithm. This improvement underscores the superiority of our modifications to the A* algorithm and indicates promising prospects for its practical application.

Compared to traditional methods, this improvement to the A* algorithm eliminates redundant path nodes and avoids excessive acceleration and deceleration operations, enhancing the robot’s stability, reducing operational wear and tear, and extending its service life, making it better suited for practical applications.

Experimental results demonstrate that, compared to the A* and DWA algorithms, the improved hybrid algorithm enhances search efficiency by 10.93%, reduces search node count by 32.26%, decreases the number of turning points by 36.36% and the value of turning angle by 34.83%, shortens the total path length by 22.05%, and improves overall path smoothness. The proposed hybrid algorithm is more stable, significantly reduces collision probability, and demonstrates its applicability for mobile robot localization and navigation in real-world environments.

By observing the robot’s path in the simulation environment and comparing it with the unoptimized path, it is evident that the improved integrated algorithm effectively reduces path length and search time while decreasing the number of nodes on the optimal path, resulting in a smoother trajectory. While the A* algorithm performs well in global static environments, it faces challenges related to high computational load in dynamic and complex local environments. Combining it with the DWA algorithm mitigates these limitations by enhancing robustness in local path planning. However, considering the impact of dynamic and static obstacles on path planning decisions in complex environments, future work will focus on further optimizing the integrated algorithm. This includes exploring 3D path planning and dynamic obstacle avoidance in hardware systems to improve adaptability and overall robustness in complex environments.

## Figures and Tables

**Table 1:** 31 × 31 Grid comparison results.

Map	Algorithm	Nodes	Costs	Turning Points	Time
Grid 1	Traditional A-star	821	1341	435	5.32
	Improved A-star	634	1034	386	4.23
Grid 2	Traditional A-star	756	1165	378	5.76
	Improved A-star	687	1067	329	4.17
Grid 3	Traditional A-star	823	1234	398	5.10
	Improved A-star	712	1123	319	3.67

**Table 2:** The comparison results of three grid maps.

Grid Map	Algorithm	Path Length	Operation Time	Searching Nodes	Path Cost	Turning Points
10% obstacles	Traditional A-star	213.13	4.67	265	376	156
	Reference	209.31	4.17	256	357	143
	Ours	201.35	3.65	234	315	125
20% obstacles	Traditional A-star	246.49	6.85	376	487	256
	Reference	235.67	6.19	356	416	242
	Ours	213.95	5.43	315	375	216
30% obstacles	Traditional A-star	258.31	7.86	467	534	276
	Reference	245.67	7.25	446	517	257
	Ours	223.57	6.56	418	478	227

**Table 3:** Simulation platform.

Platform	Parameter	Value
Hardware	CPU	Intel(R)CoreTM i3-12100F 3.30 GHz (Intel, Mountain View, CA, USA)
	GPU	Nvidia GeForce RTX 4060 (Nvidia, Santa Clara, CA, USA)
	RAM	16 GB
Software	OS	Ubuntu 20.04
	ROS	Noetic
	Gazebo	11.11

**Table 4:** Output data of various comparative parameters.

Environment Map	Algorithm	Search Time/s	Number of Nodes	Turning Points	Turning Angle/°	Path Length/cm
Road	A*	42.47	31	11	178	45.12
	Ours	37.83	21	7	116	35.17

**Table 5:** The results of parameter comparison.

Map	Algorithm	Length	Angle	Turning Points	Time	Avoid Dynamic Obstacle
30 × 30	Reference	36.78	178°	66	196.99 s	failure
	Ours	28.67	148°	47	169.46 s	success

## Data Availability

The data analyzed in the course of this study are not publicly available but can be obtained from the corresponding authors upon reasonable request.

## Abbreviations

The following abbreviations are used in this manuscript:

DWA	Dynamic Window Approach

## References

[1] Han D., Jung S., Lee M., Kim J., Park C. (2014). Building a practical Wi-Fi-based indoor navigation system. IEEE Pervasive Comput..

[2] Jiang S., Sun S., Li C. (2024). Path planning for outdoor mobile robots based on IDDQN. IEEE Access.

[3] Yang F., Fang X., Gao F., Zhou X., Li H., Jin H., Song Y. (2022). Obstacle avoidance path planning for UAV based on improved RRT algorithm. Discret. Dyn. Nat. Soc..

[4] Li X., Yu S., Gao X., Yan Y., Zhao Y. (2024). Path planning and obstacle avoidance control of UUV based on an enhanced A* algorithm and MPC in dynamic environment. Ocean Eng..

[5] Ntakolia C., Moustakidis S., Siouras A. (2023). Autonomous path planning with obstacle avoidance for smart assistive systems. Expert Syst. Appl..

[6] Wang H., Lou S., Jing J., Wang Y., Liu W., Liu T. (2022). The EBS-A* algorithm: An improved A* algorithm for path planning. PLoS ONE.

[7] Liao T., Chen F., Wu Y., Zeng H., Ouyang S., Guan J. (2024). Research on path planning with the integration of adaptive A-star algorithm and improved dynamic window approach. Electronics.

[8] Zhang H., Tao Y., Zhu W. (2023). Global path planning of unmanned surface vehicle based on improved A-Star algorithm. Sensors.

[9] Li Y., Li G., Wang X. (2024). Research on Trajectory Planning of Autonomous Vehicles in Constrained Spaces. Sensors.

[10] Xu B. (2024). Precise path planning and trajectory tracking based on improved A-star algorithm. Meas. Control.

[11] Zeng X., Zhang J., Yin W., Yang H., Yu H., Liang Y., Tong J. (2024). Path planning strategies for logistics robots: Integrating enhanced A-star algorithm and DWA. Electron. Lett..

[12] Chen Z., Zhang Y., Zhang Y., Li Y., Wang J. (2019). A hybrid path planning algorithm for unmanned surface vehicles in complex environment with dynamic obstacles. IEEE Access.

[13] Kumar S., Parhi D.R., Kashyap A.K., Pradhan S.K. (2021). Static and dynamic path optimization of multiple mobile robot using hybridized fuzzy logic-whale optimization algorithm. Proc. Inst. Mech. Eng. Part C J. Mech. Eng. Sci..

[14] Akay R., Yildirim M.Y. (2023). Multi-strategy and self-adaptive differential sine–cosine algorithm for multi-robot path planning. Expert Syst. Appl..

[15] Sheng W., Li B., Zhong X. (2021). Autonomous parking trajectory planning with tiny passages: A combination of multistage hybrid A-star algorithm and numerical optimal control. IEEE Access.

[16] Meng T., Yang T., Huang J., Zhang X., Li H. (2023). Improved hybrid A-star algorithm for path planning in autonomous parking system based on multi-stage dynamic optimization. Int. J. Automot. Technol..

[17] Dang C.V., Ahn H., Lee D.S., Kim J. (2022). Improved analytic expansions in hybrid A-star path planning for non-holonomic robots. Appl. Sci..

[18] Noto M., Sato H. A method for the shortest path search by extended Dijkstra algorithm. Proceedings of the 2000 IEEE International Conference on Systems, Man and Cybernetics.

[19] García A. (2024). Greedy algorithms: A review and open problems. arXiv.

[20] Palacín J., Rubies E., Bitriá R., Martínez C. (2023). Path Planning of a Mobile Delivery Robot Operating in a Multi-Story Building Based on a Predefined Navigation Tree. Sensors.

[21] Albert B., Antoine V., Koko J. (2024). ECM+: An improved Evidential c-means with adaptive distance. Fuzzy Sets Syst..

[22] Fransen K., van Eekelen J. (2023). Efficient path planning for automated guided vehicles using A* algorithm incorporating turning costs in search heuristic. Int. J. Prod. Res..

[23] Lai R., Wu Z., Liu X., Chen Z. (2023). Fusion algorithm of the improved A* algorithm and segmented Bezier curves for the path planning of mobile robots. Sustainability.

[24] Huang J., Chen C., Shen J., Liu G., Xu F. (2025). A self-adaptive neighborhood search A-star algorithm for mobile robots global path planning. Comput. Electr. Eng..

[25] Chung M.A., Lin C.W. (2021). An improved localization of mobile robotic system based on AMCL algorithm. IEEE Sens. J..

[26] Huo L., Liu Y., Yang Y., Chen J. (2023). Research on product surface quality inspection technology based on 3D point cloud. Adv. Mech. Eng..

[27] Li Q., Xue Y. (2023). Total leaf area estimation based on the total grid area measured using mobile laser scanning. Comput. Electron. Agric..

[28] Varanasi S.D., Tammana M., Megalingam R.K. Robotic Navigation Unveiled: A Comprehensive Study of GMapping, Hector Slam, and Cartographer. Proceedings of the 2024 3rd International Conference for Innovation in Technology (INOCON).

[29] Ghazal M.T., Al-Ghadhanfari M., Waisi N.Z. (2024). Simulation of autonomous navigation of TurtleBot robot system based on robot operating system. Bull. Electr. Eng. Inf..

[30] Chen H. (2022). Development of Teaching Material for Robot Operating System (ROS): Creation and Control of Robots. Master’s Thesis.

[31] Nocito M. (2024). URDF Studio: Tools for the Visualization and Verification of Universal Robot Description Format. Master’s Thesis.

[32] Schneider D., Kastner L., Schick B., Rohde F. (2024). fROS: A Generic Fieldbus Framework for ROS. IEEE Trans. Intell. Veh..

[33] Li C., Wang S., Zhuang Y., Tang J. (2019). Deep sensor fusion between 2D laser scanner and IMU for mobile robot localization. IEEE Sens. J..

[34] Dong D., Dong S., Zhang L., Cai Y. (2024). Path Planning Based on A-Star and Dynamic Window Approach Algorithm for Wild Environment. J. Shanghai Jiaotong Univ..

